# Morphological and molecular evidence reveals a new species of *Characidium* from the Ucayali‐Urubamba Piedmont, Peru, and novel molecular clades are proposed within the genus

**DOI:** 10.1111/jfb.70261

**Published:** 2025-11-06

**Authors:** Leonardo Oliveira‐Silva, Ricardo Britzke, Vanessa Meza‐Vargas, Max H. Hidalgo, Dario Faustino‐Fuster, Claudio Oliveira, Angela Maria Zanata

**Affiliations:** ^1^ Departamento de Biologia Estrutural e Funcional Instituto de Biociências, Universidade Estadual Paulista São Paulo Brazil; ^2^ Department of Ichthyology American Museum of Natural History New York New York USA; ^3^ Museo de Historia Natural Universidad Nacional Mayor de San Marcos Lima Peru; ^4^ Facultad de Ciencias Biológicas Universidad Nacional Mayor de San Marcos Lima Peru; ^5^ Programa de Pós‐Graduação em Biodiversidade e Evolução, Instituto de Biologia Universidade Federal da Bahia Salvador Brazil

**Keywords:** Amazon fishes, Andean piedmont, freshwater biodiversity, Neotropical fishes, species delimitation

## Abstract

A new species of *Characidium* is described from the Urubamba River basin, within the Ucayali‐Urubamba Piedmont ecoregion, Peru. The new species can be readily distinguished from all congeners, except *Characidium cacah*, *Characidium chicoi*, *Characidium helmeri*, *Characidium mirim*, *Characidium nana*, *Characidium nupelia*, *Characidium sterbai*, *Characidium stigmosum* and *Characidium xavante* by the possession of an incomplete lateral line and the lack of an adipose fin. It differs from these species, and remaining congeners, by a unique sexually dimorphic colour pattern: females exhibit irregular dark blotches along the dorsum that alternate elongation between the sides of the body and are usually disconnected from the lateral bars, whereas in males the dark bars are absent. Additional diagnostic features include two series of dentary teeth, absence of a dark blotch on the caudal peduncle, 12 circumpeduncular scales and a fully scaled isthmus. Molecular data indicate a significant genetic divergence between the new species and its closest relatives, further confirming its recognition as a distinct species. Additionally, two new clades were recovered in molecular phylogeny, each formed mostly by species not previously included in any molecular phylogenetic studies.

## 1 INTRODUCTION

The genus *Characidium*, member of the family Crenuchidae, is renowned for its taxonomic richness and diversity, extensively distributed across freshwater streams and rivers in South America (Zanata et al., 2024; Toledo‐Piza et al., 2024; Fricke et al., 2025). The genus comprises 87 valid species characterized by adaptations to a wide range of habitats, from fast‐flowing mountain streams to slower lowland waters (Fricke et al., 2025; Oliveira‐Silva et al., 2024). Distinctive morphological features, such as variations in fin structures and colouration, reflect the adaptive diversity within the genus (Melo et al., 2016; Zanata et al., 2020; Zanata et al., 2023). Taxonomic studies have been crucial in the delimitation of species of *Characidium*, employing both traditional morphological characteristics and molecular analyses to elucidate their complex phylogenetic relationships (e.g., Oliveira‐Silva et al., 2024; Serrano et al., 2018). The ongoing description of new species by a series of authors (e.g., Armbruster et al., 2021; Stabile et al., 2025; Zanata et al., 2024) highlights that the diversity of the genus is still far from fully known.

The Ucayali‐Urubamba Piedmont ecoregion (UUP‐Freshwater Ecoregions of the World; Abell et al., 2008) is one of Peru's main rivers, representing approximately 5% of the Andean‐Amazon basin (WCS, 2021). Eight species of *Characidium* are currently known to the UUP: *Characidium etheostoma* Cope 1872, *Characidium geryi* (Zarske 1997), *Characidium pteroides* Eigenmann 1909, *Characidium purpuratum* Steindachner 1882, *Characidium roesseli* Géry 1965, *Characidium steindachneri* Cope 1878, *Characidium sterbai* (Zarske 1997) and *Characidium zebra* Eigenmann 1909 (Chuctaya et al., 2022). A previous survey of the fish fauna in the Urubamba River sub‐basin, a tributary of the Ucayali River, revealed populations of *Characidium* with uncertain identification (e.g., *Characidium* sp. 1, *Characidium* sp. 2), underscoring the limited taxonomic knowledge of the genus in the region (Carvalho et al., 2011). Furthermore, records from scientific collections based on fieldwork in the basin indicate the occurrence of undescribed populations (*Characidium* sp.) or those morphologically similar to known species (e.g., *C*. aff. *purpuratum*, *C*. aff. *zebra*), which still awaits formal taxonomic delimitation. Thus, the species richness of *Characidium* in the region is likely greater than currently recognized. The present study supports this hypothesis by describing a new species of *Characidium* from the Cusco region, Urubamba River basin, discovered recently through a brief taxonomic assessment of specimens housed in the Museo de Historia Natural, Lima, Peru. The description is based on an integrative taxonomic approach that combines both morphological and molecular data. Additionally, we propose novel clades within *Characidium* and briefly discuss the phylogenetic placement of the new species as inferred from the molecular results.

## 2 MATERIALS AND METHODS

### 2.1 Morphological analyses

Counts and measurements were conducted according to Buckup (1993a), Melo and Oyakawa (2015) and Zanata et al. (2020), except for the dorsal to adipose fin distance, which was omitted as the new species lacks an adipose fin. Measurements were obtained using a digital calliper, accurate to 0.1 mm, and are presented as percentage of standard length (*L*_*S*_), except for head subunits, which are expressed relative to head length (*L*_*H*_). In the list of paratypes, an asterisk (*) identifies lots with specimens used for measurements and meristic data, and the specimens used in molecular analyses are indicated with ‘mol’. Meristic characters are given in the species description, with holotype values marked by an asterisk and the frequency of each count noted in parentheses. Vertebrae, dentary and ectopterygoid teeth, branchiostegal rays, procurrent caudal‐fin rays, epurals and additional osteological data were gathered exclusively from cleared and stained (c&s) paratypes, processed following Taylor and Van Dyke (1985) protocol. Scale morphology, including *circuli* and *radii* patterns, was analysed from scales located between the dorsal‐fin origin and the lateral line, after staining with alizarin. The structure of the pseudotympanum was examined by removing the skin, adipose tissue and lateral‐line nerve from alcohol‐preserved specimens. Institutional abbreviations are consistent with those listed by Fricke and Eschmeyer (2025).

### 2.2 DNA amplification and sequencing

The animals in this research, whose samples were included and processed at Laboratório de Genética de Peixes, UNESP, Botucatu (LBP), were used in accordance with Brazilian animal welfare laws. This study was approved by the Ethics Committee on Animal Use of the Biosciences Institute of UNESP (licence number: CEUA3337150524).

Genomic DNA was extracted from muscle tissue using a Wizard Genomic DNA Purification Kit (Promega; www.promega.com), following the manufacturer's guidelines (paratypes used in molecular analyses are identified as ‘mol’.). Polymerase chain reaction (PCR) amplifications were performed in a final volume of 12.5 μL, which included 1.25 μL of 10× reaction buffer, 0.25 μL of MgCl₂ (50 mM), 0.2 μL of dNTPs (2 mM), 0.5 μL of each primer (5 mM), 0.1 μL of PHT Taq DNA polymerase (Phoneutria), 1.0 μL of genomic DNA (200 ng) and 8.7 μL of ultrapure water (ddH₂O). The mitochondrial marker cytochrome oxidase subunit I (coI) was amplified using primers from Ward et al. (2005). The thermal cycling protocol included an initial denaturation step (5 min at 94°C), followed by 30 cycles of strand denaturation (50 s at 94°C), primer annealing (45 s at 50–54°C) and DNA elongation (1 min at 68°C), with a final extension phase (10 min at 68°C). All PCR products were checked on 1% agarose gels and then purified using ExoSap‐IT (USB Corporation) according to the manufacturer's instructions. The purified PCR products were subjected to sequencing procedures with the BigDye Terminator version 3.1 Cycle Sequencing Ready Reaction Kit (Applied Biosystems) and purified by ethanol precipitation. Finally, the sequencing reactions were analysed on an ABI 3130 DNA Analyser automated sequencer (Applied Biosystems).

### 2.3 Molecular data analysis

In the present study, new coI sequences were generated, including two sequences attributed to the newly identified species, as well as sequences from other *Characidium* species for which no sequences were previously available. These include *Characidium amaila* Lujan, Agudelo‐Zamora, Taphorn, Booth & López‐Fernández 2013, *Characidium brevirostre* Pellegrin 1909, *Characidium fleurdelis* Zanata, Oliveira‐Silva & Ohara 2023, *Characidium hasemani* Steindachner 1915, *Characidium laterale* (Boulenger 1895), *Characidium longum* Taphorn, Montaña & Buckup 2006, *Characidium nupelia* Graça, Pavanelli & Buckup 2008, *Characidium occidentale* Buckup & Reis 1997, *Characidium papachibe* Peixoto & Wosiacki, 2013, *Characidium pumarinri* Teixeira & Melo 2020, *Characidium roesseli* and *C. sterbai*. Additionally, sequences from prior investigations involving *Characidium* were incorporated (e.g., Agudelo‐Zamora et al., 2020; Oliveira‐Silva et al., 2024; Serrano et al., 2018; Stabile et al., 2025; Zanata et al., 2024), which are accessible at GenBank. We utilized a sequence of *Crenuchus spilurus* Günther 1863 from GenBank to serve as the root for the phylogenetic tree. For more details about sequences and GenBank numbers, see Table S1. The sequences were assembled using the software Geneious 7.1.4 (Kearse et al., 2012), and aligned with Muscle (Edgar, 2004) under default parameters. After alignments, the matrix was checked visually in the MEGA version 11 (Tamura et al., 2021) for manual corrections if necessary, and sequences were validated by their translation into amino acids. The substitution saturation index (Iss) was determined using DAMBE version 5.3.38 (Xia, 2013).

Maximum likelihood (ML) analysis was carried out using RAxML PTHREADS‐SSE3 version 8 (Stamatakis, 2014) with five random parsimony trees and the GTRGAMMA model on the *Zungaro* server at LBP‐UNESP. The best tree was obtained through 10 random searches with 1000 bootstrap pseudoreplicates using the autoMRE function with bootstrapping criteria (Pattengale et al., 2010). Assemble Species by Automatic Partitioning (ASAP; Puillandre et al., 2021) and Bayesian implementation of the Poisson Tree Process model (bPTP; Zhang et al., 2013) were employed to assess the molecular validity of the new species. ASAP analysis was conducted via the web server (https://bioinfo.mnhn.fr/abi/public/asap/asapweb.html) utilizing the Kimura (K80; 2.0) model, whereas bPTP utilized the best ML tree as input, underwent 100,000 generations and employed default parameters on the bPTP web server (http://species.h-its.org/ptp/). The analyses of ASAP and bPTP excluded the out‐group taxa. Overall and pair‐wise genetic distances were estimated using the p‐distance model (K2P) + Gamma in MEGA version 11. The analyses of ASAP, bPTP and genetic distances were calculated using only the alignment containing the sequences belonging to the proposed New Clade I (see result session).

## 3 RESULTS

### 3.1 *Characidium ortegai*, new species

urn:lsid:zoobank.org:pub:E4854AB7‐BDD9‐40B9‐A878‐B5F73C868C56.

urn:lsid:zoobank.org:act:29B6DB0B‐6449‐43FA‐BEC1‐6B937E9087B3.

**FIGURE 1:**
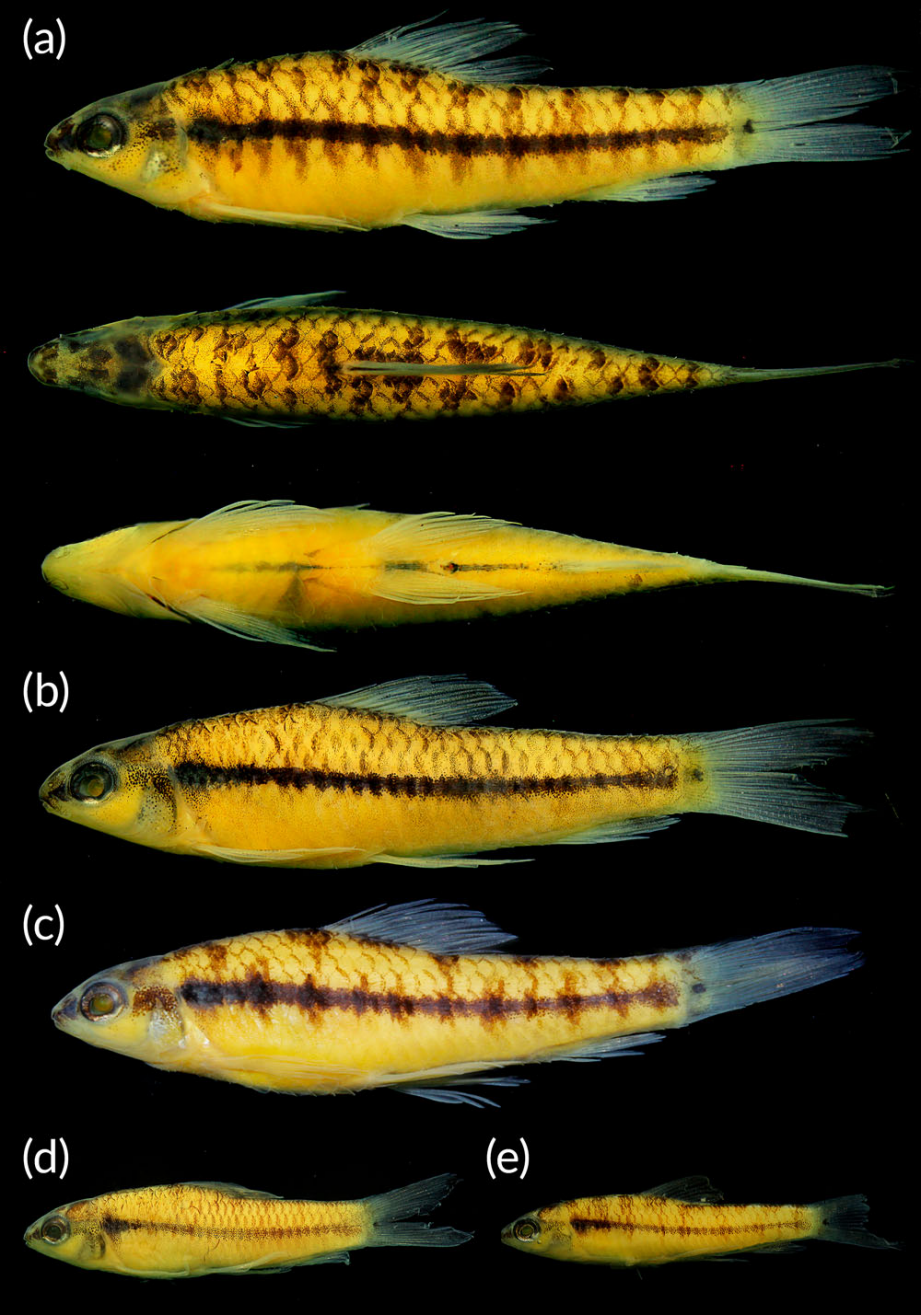
*Characidium ortegai*: (a) holotype, MUSM 77985, 30.2 mm standard length (SL), lateral, dorsal and ventral views; (b) paratype, MZUSP 131440, male, 32.3 mm SL; (c) paratype, MUSM 70838, female, 31.0 mm standard length; (d) paratype, MUSM 44939, male, 22.9 mm SL; (e) paratype, MUSM 44939, female, 21.8 mm SL. All from Urubamba drainage, Ucayali River sub‐basin, Amazon River basin, Peru, Cusco Department, La Convención Province, Megantoni District.

**FIGURE 2:**
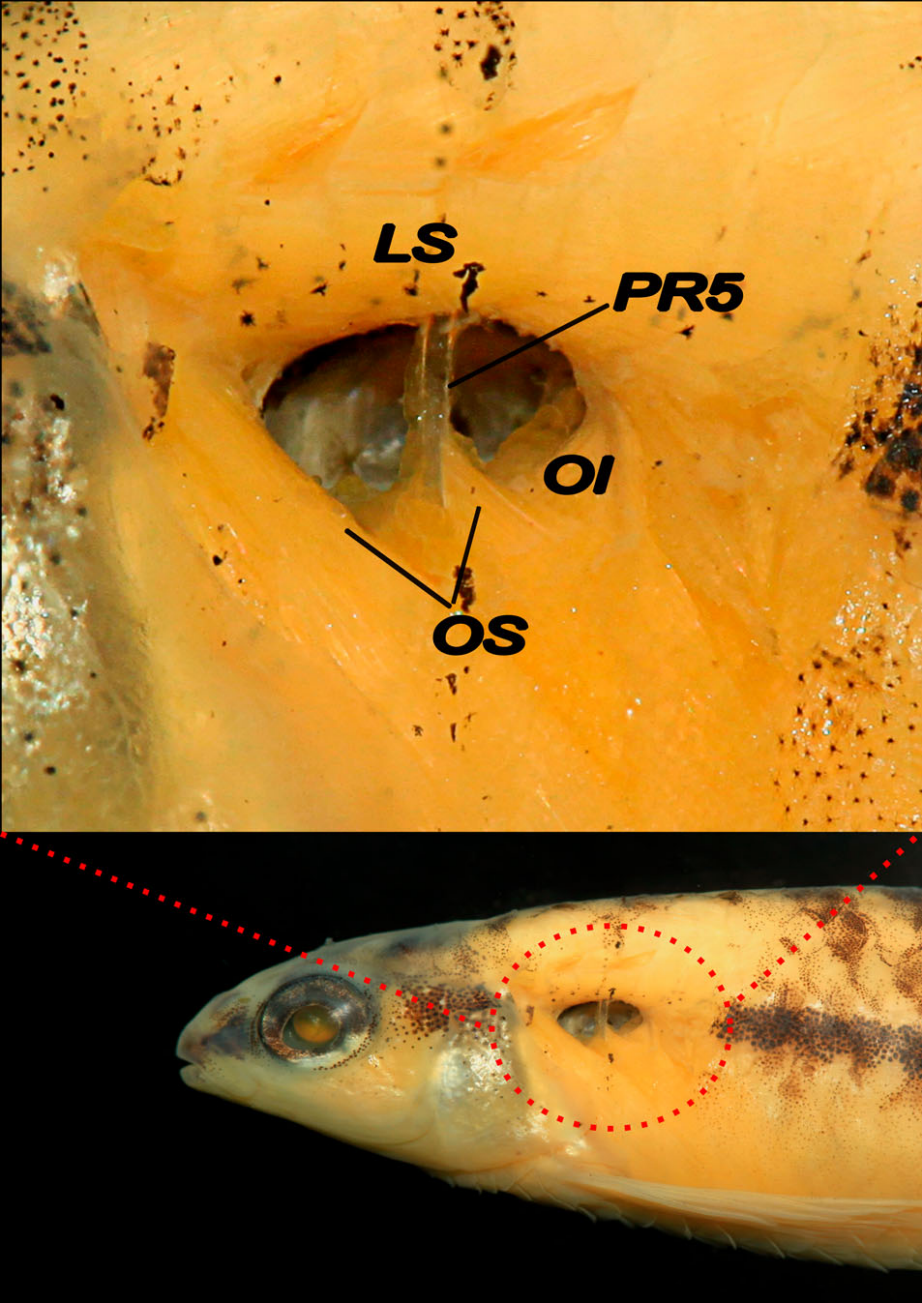
Pseudotympanum of *Characidium ortegai* (MUSM 70838, 34.0 mm standard length, paratype) in right lateral view. Overlying skin, adipose tissue and lateral‐line nerve removed. (OS) *obliquus superioris*; (OI) *obliquus inferioris*; (*LS*) *lateralis superficialis*; (PR5) pleural rib of the fifth vertebrae.

Figures 1 and 2, Table 1.

**TABLE 1:** Morphometric data of holotype and paratypes of *Characidium ortegai* (*n* = 25), range includes the holotype.

	Holotype	Range	Mean	SD
Total length (mm)	38.9	31.9–43.8	–	–
Standard length (mm)	30.2	24.4–34.8	–	–
Percentage of standard length				
Depth at dorsal‐fin origin	22.8	22.5–26.6	24.2	1.2
Depth at anal‐fin origin	16.2	14.9–18.0	16.9	0.9
Caudal‐peduncle depth	11.9	10.6–13.5	12.3	0.9
Caudal‐peduncle length	15.2	14.1–17.6	16.0	0.9
Snout to dorsal‐fin origin	44.4	43.1–47.6	45.1	1.1
Snout to pectoral‐fin origin	24.8	23.2–26.3	24.9	0.8
Snout to pelvic‐fin origin	52.0	49.8–52.7	51.4	0.9
Snout to anal‐fin origin	78.1	75.9–79.3	77.7	0.9
Anal‐apex distance	96.4	94.7–98.1	96.3	0.9
Dorsal‐fin adpressed	28.8	28.2–31.3	29.5	0.9
Pectoral‐fin length	23.8	23.8–27.0	25.3	0.9
Pelvic‐fin length	23.5	21.9–26.4	24.0	1.0
Anus to anal‐fin origin	15.9	13.7–16.6	15.2	0.8
Body width	10.5	10.2–15.0	11.8	1.2
Head length	23.2	22.9–25.2	23.8	0.9
Percentage of head length				
Horizontal eye diameter	30.0	29.3–32.3	30.8	0.9
Snout length	20.0	19.5–23.3	21.6	1.0
Snout to maxillary tip	25.7	22.2–25.7	23.8	0.9
Anterior naris to orbit	10.0	8.1–10.3	9.5	0.5
Posterior naris to orbit	4.3	3.1–5.6	4.9	0.7
Cheek depth	5.7	3.1–7.8	5.8	1.2
Least interorbital width	25.7	22.6–26.2	22.8	1.0

Abbreviation: SD, standard deviation.

*Geryichthys sterbai*. – Carvalho et al. (2011): p. 4166 (Fishes from the Lower Urubamba) second photo in p. 421.

### 3.2 Holotype

MUSM 77985, 30.2 mm *L*_*S*_, Peru, Cusco Department, La Convención Province, Megantoni District, Charapa creek tributary of Urubamba River, Ucayali River sub‐basin, Amazon River basin, 11°21′5.49″ S, 73°0′25.35″ W, A. Mendoza 24 Oct 2022 (Figure 1a).

### 3.3 Paratypes

**All from Peru, Cusco Department, La Convención Province, Megantoni District, Ucayali River sub‐basin, Urubamba drainage**. MUSM 12005, 1, 31.4 mm *L*_*S*_, Maseriato Creek tributary of Parotori River, 12°05′60.00″ S, 73°09′0.00″ W, 20 May 1997, F Chang. MUSM 12039, 7, 16.3–29.7 mm *L*_*S*_, Cocha Kamariampiveni, 11°46′48.00″ S, 73°09′0.00″ W, 22 May 1997, F. Chang. MUSM 12118, 3, 31.1–33.2 mm *L*_*S*_, Mayapo River tributary of Picha River, 11°34′48.01″ S, 73°07′48.02″ W, 25 May 1997, F. Chang. MUSM 23034, 4, 28.3–31.5 mm *L*_*S*_, Shimbillo Creek tributary of Urubamba River, 11°21′43.14″ S, 73°00′11.37″ W, 22 Sep 2004, H. Ortega. MUSM 36115, 3, 25.5–29.2 mm *L*_*S*_, Piriabindeni Creek, tributary of Parotori River, 12°01′14.52″ S, 73°03′51.82″ W, 28 May 2009, R. Quispe. MUSM 36131, 3, 23.8–32.0 mm *L*_*S*_, Pariabindeni Creek tributary of Parotori River, 12°01′19.29″ S, 73°04′14.66″ W, 28 May 2009, R. Quispe. MUSM 38390, 4, 29.4–32.7 mm *L*_*S*_, Charapa Creek, tributary of Miaria River, 11°21′05.23″ S, 73°00′25.69″ W, 19 Sep 2019. MUSM 41752*, 6, 29.0–30.7 mm *L*_*S*_, Charapa Creek tributary of Miaria River, 11°20′36.46″ S, 73°00′24.93″ W, 20 Aug 2011, I. Sipión. MUSM 44939, 13, 18.9–28.2 mm *L*_*S*_, unnamed creek tributary of Urubamba River, Nuevo Mundo, 11°32′16.76″ S, 73°09′14.46″ W, 8 May 2012, O. Nash. MUSM 48978, 2, 27.5–30.8 mm *L*_*S*_, Maputonoari Creek tributary of Picha River, 11°49′12.80″ S, 73°11′11.14″ W, 8 Jul 2014, M. Armas. MUSM 49258, 1, 28.2 mm *L*_*S*_, Sensa River tributary of Urubamba River, 11°24′38.50″ S, 73°21′39.36″ W, 11 Mar 2014, A. Meza. MUSM 52674, 3, 23.7–30.0 mm *L*_*S*_, Parauinteni Creek tributary of Picha River, 12°01′42.85″ S, 73°05′30.52″ W, 19 Jun 2015, S. Valenzuela & R. Ricce. MUSM 53061, 1, 29.4 mm *L*_*S*_, unnamed creek tributary of Picha River, 11°48′41.83″ S, 73°10′06.93″ W, 01 Sep 2015, N. Faustino. MUSM 69901*, 11, 25.4–33.0 mm *L*_*S*_, Niantoari Creek tributary of Picha River, 11°49′46.98″ S, 73°11′18.42″ W, 20 Sep 2021, F. Cari. MUSM 69912*, 6, 30.6–31.9 mm *L*_*S*_, Maputonoari Creek tributary of Picha River, 11°48′41.64″ S, 73°10′07.07″ W, 20 Sep 2021, F. Cari. MUSM 70838*, 22, 28.3–34.6 *L*_*S*_, same data as holotype. MUSM 77986, 3, 21.99–27.59 mm *L*_*S*_, mol, Charapa Creek, tributary of Urubamba River, 11°51′11.08″ S, 72°57′10.38″ W, 2 Aug 2024, G. Valenzuela. LBP 37171, 5, 29.0–30.8 mm *L*_*S*_; same data as holotype. MZUSP 131440, 5, 29.3–31.4 mm *L*_*S*_, same data as holotype. UFBA 10830, 7, 27.8–32.0 mm *L*_*S*_, 2 c&s, female 32.0 mm *L*_*S*_ and male 29.2 mm *L*_*S*_, same data as holotype.

### 3.4 Diagnosis

*C. ortegai* can be distinguished from all congeners, except *C*. *cacah* Zanata, Ribeiro, Araújo‐Porto, Pessali & Oliveira‐Silva, *C. chicoi* Graça, Ota & Domingues 2019, *C. helmeri* Zanata, Sarmento‐Soares & Martins‐Pinheiro 2015, *C. mirim* Netto‐Ferreira, Birindelli & Buckup 2013, *C. nana* Mendonça & Netto‐Ferreira 2015, *C. nupelia*, *C. sterbai*, *C. stigmosum* Melo & Buckup 2002, and *C. xavante* Graça, Pavanelli & Buckup 2008, by the possession of an incomplete lateral line and the lack of the adipose fin (vs. lateral line complete and adipose‐fin present). The new species is diagnosed from the aforementioned species by its colour pattern, with females having irregular dark blotches along body dorsum, which alternate in elongation to one or other side of body and are usually not connected to lateral blotches or bars and males without dark blotches or bars on flanks (vs. when present, dorsum blotches and lateral bars connected). Additionally, *C. ortegai* differs from *C. cacah, C. chicoi*, *C. mirim*, *C. nupelia*, *C. stigmosum* and *C. xavante* by the possession of two series of dentary teeth (vs. a single series of dentary teeth). The new species can be further distinguished from *C. helmeri* and *C. stigmosum* by having 12 circumpeduncular scales (vs. 13 or 14), from *C. nana, C. nupelia* and *C. xavante* by lacking a dark horizontally elongate blotch on the caudal peduncle (vs. blotch present). It can be further distinguished from *C. helmeri* and *C. sterbai* by having the isthmus completely covered by scales (vs. anteriormost portion of isthmus naked). Additionally, *C. ortegai* differs from *C. helmeri* by having well‐developed supraorbitals (vs. supraorbitals reduced or absent), and from *C. sterbai* by having a distinctly posteriorly positioned parietal fontanel, limited anteriorly by the parietals (vs. parietal fontanel reaching anteriorly, anteriorly limited by the frontals), dentary and premaxilla with tricuspid and unicuspid teeth (vs. only conical teeth present). Besides morphological and meristic characters, *C. ortegai* can be distinguished from the congeners by molecular divergence (see Results and Discussion for more details regarding morphological and molecular differences).

### 3.5 Description

Holotype and 24 paratypes with morphometric data are presented in Table 1. Body elongate and moderately compressed. Greatest body depth at vertical through dorsal‐fin origin. Dorsal profile convex from upper lip to interorbital area, slightly convex from this point to dorsal‐fin origin, convex and posteroventrally inclined along dorsal‐fin base, almost straight between dorsal‐fin terminus to anteriormost caudal‐fin procurrent ray. Ventral profile slightly convex near dentary symphysis, straight or slightly convex from that area to pelvic‐fin origin, straight from latter point to anal‐fin origin, slightly concave along anal‐fin base, and straight from anal‐fin terminus to anteriormost ventral caudal‐fin procurrent ray. Snout triangular in lateral view, rounded in dorsal view. Mouth terminal or slightly subterminal, anterior borders of teeth usually visible in ventral view. Distal tip of maxilla not reaching or barely reaching vertical through anterior margin of orbit. Orbit approximately round, distinctly larger than snout. Cheek narrow, its depth shorter than third of orbit diameter. Nares separated and without distinctly raised margins, posterior naris considerably closer to orbit than to anterior naris. Supraorbital well developed, border abutting frontal convex and outer border slightly concave. Nasal bones restricted to ossified canal. Cranial fontanel located distinctly posteriorly, close to posterior border of parietals, restricted to somewhat triangular or half‐circle shaped opening between contralateral parietals. Parietal branch of supraorbital canal absent.

Dentary teeth in two rows: outer row with 11(2) teeth, uni‐ or tricuspid, with lateral cusps distinctly small and hardly visible; teeth decreasing in size and number of cusps from symphysis; last three or four teeth unicuspid. Premaxilla with single row of 8(2) teeth similar to those on dentary, decreasing slightly in size from symphysis; last teeth unicuspid. Maxilla edentulous. Ectopterygoid with 5(1) or 6(1) teeth arranged in one (2) row. Endopterygoid teeth absent. Branchiostegal rays 5(2), 4 connected to anterior ceratohyal, 1 connected to posterior ceratohyal.

Scales cycloid; *circuli* absent on exposed portion of scales; up to 8 divergent *radii* on exposed portion of scales of largest specimens. Lateral line reduced; perforated scales 8(4), 9*(14) or 10(7); total scales along lateral line series 30(9), 31(3), 32*(9) or 33(4); horizontal scale rows above lateral line 4*(25); horizontal scale rows from lateral line to midventral scale series 4*(25). Predorsal scales 8*(3), 9(14), 10(6) or irregularly arranged (2). Scale rows around caudal peduncle 12(25). Four*(3), 5(19) or 6(3) scales between anus aperture and anal‐fin insertion. Isthmus and belly completely covered by scales. Pseudotympanum appears as muscular hiatus, bordered dorsally by *lateralis superficialis*, anteriorly and posteriorly by *obliquus inferioris* and ventrally by *obliquus superioris*. Humeral hiatus divided into anterior and posterior chambers by pleural rib of fifth vertebra (Figure 2). Swimbladder long, somewhat pointed posteriorly; total length 15.7%–16.0% of *L*_*S*_ (2 specimens, 29.2–32.0 mm *L*_*S*_).

Dorsal‐fin rays iii,9(5), or ii,10*(20); distal margin of fin nearly straight or somewhat rounded. First dorsal‐fin radial inserting behind 9th(2) vertebrae. Adipose fin absent. Pectoral‐fin with 9–11 total rays; iii,5,i(7), iii,6,i*(17) or iii,7,i(1); second and third branched pectoral‐fin rays usually longest; posterior tip of pectoral fin reaching or almost reaching pelvic‐fin insertion. Postcleithrum 1 absent; 2 and 3 present. Pelvic‐fin rays i,7,i*(25); second to fourth branched pelvic‐fin rays longest; posterior tip of pelvic fin falling short of anal‐fin origin. Anal‐fin rays ii,6*(25); posterior margin of anal fin straight or slightly rounded, with first and second branched rays usually longest. First anal‐fin radial inserting behind 21st(1) vertebra or 22th(1) vertebra, behind 5th(1) or 7th(1) caudal vertebra; fin elements on last pterygiophore 2(2). Caudal‐fin rays i,7,7,i (1) or i,8,7,i*(24). Dorsal procurrent caudal‐fin rays 7(2); ventral procurrent caudal‐fin rays 6(2). Total vertebrae 31(1) or 32 (1); precaudal vertebrae 11(1) or 12(1); caudal vertebrae 15(1) or 17(1). Supraneural bones 4(2). Epural bones 2(2). Uroneural bone 1(2).

### 3.6 Colour in alcohol

*Female* (Figure 1a,c). Ground colour of head and body pale yellow or pale brown. Dorsal surface of head dark, with broad clear area between anterior portion of eyes and tip of snout. In lateral view, dorsal half of head darker than ventral half, with dark stripe from anterior margin of snout to anterior margin of eye, aligned with relatively diffuse blotch posterior to eye. Ventral half of head without melanophores, except by dark border of inferior lip and area of maxillary bone visible in ventral view. Dorsum of body with up to 13 saddled blotches, more conspicuous than lateral ones; dorsal blotches alternating elongation to one or other side of body mainly on predorsal area, but usually not continuous with lateral blotches; dorsal blotches more regularly elongated to both sides of body posteriorly. Conspicuous dark narrow stripe, around one scale wide, extending from rear of opercle to end of caudal peduncle; portion of stripe immediately posterior to humeral blotch darker and more conspicuous than remaining extent of stripe in some specimens (Figure 1e). Humeral blotch rounded or somewhat vertically elongated; usually merged with midlateral stripe and not very distinguishable. Midlateral stripe usually crossed by narrow vertically elongated dark regular bars or irregular blotches, usually not connected to middorsal blotches (Figure 1c); bars varying from 7 to 13, but some specimens with blotches not well delimited from each other, or inconspicuous, making counting difficult. Ventral portion of blotches barely exceeding midlateral stripe and not reaching ventral half of body, except small specimens, around 18.0–25.0 mm *L*_*S*_. Basicaudal spot well marked. Ventral half of body with scattered tiny melanophores, diminishing towards ventral surface. Ventral surface of body pale yellow with few, small and scattered chromatophores. Deep dark midventral line usually present ahead of origin of pelvic fins and always present between pelvic‐fin insertion and anal‐fin origin (Figure 1a). Paired fins usually hyaline or with tiny melanophores along borders of few rays; dorsal and caudal‐fin rays with tiny melanophores along its borders, forming delicate dark lines bordering rays; bifurcation of dorsal‐fin rays well marked in black. Dorsal fin crossed by two inconspicuous dark bands formed by sparse melanophores on rays and interradial membranes; one band below midlength of rays and second on its distal portion. Anal fin with delicate dark lines bordering some rays and inconspicuous dark band crossing distal half of fin, similar to pattern present on dorsal fin.

*Male* (Figure 1b,d). Overall head, background and fins colouration similar to described for females. Dorsum homogeneously darkened or with inconspicuous blotches, not distinctly elongated laterally. Dark midlateral stripe similar to that of females, around one scale wide. Humeral dark blotch merged with stripe, somewhat vertically expanded when visible. Flanks without dark bars. Dorsal half of body distinctly darker than ventral half, with melanophores concentrated on margins of scales, resulting in a reticulate pattern. Basicaudal black spot well marked. Ventral half of body similar to females, except by absence of ventral portion of blotches crossing midlateral stripe. Midventral line with dark interrupted line equal to females.

### 3.7 Sexual dimorphism

No hooks on fins were observed. Sexual dichromatism is mainly evident on the flanks: females exhibit a dark midlateral stripe often crossed by narrow, vertically elongated bars or blotches, as well as well‐defined dorsal blotches (Figure 1a,c); in contrast, males lack these vertical blotches, displaying unmarked flanks, less conspicuous mid‐dorsal blotches and a more pronounced reticulate pattern on the dorsal half of the body due to melanophores concentrated along the scale margins (Figure 1b,d). For further details, see the Colouration in Alcohol section.

### 3.8 Distribution

**FIGURE 3:**
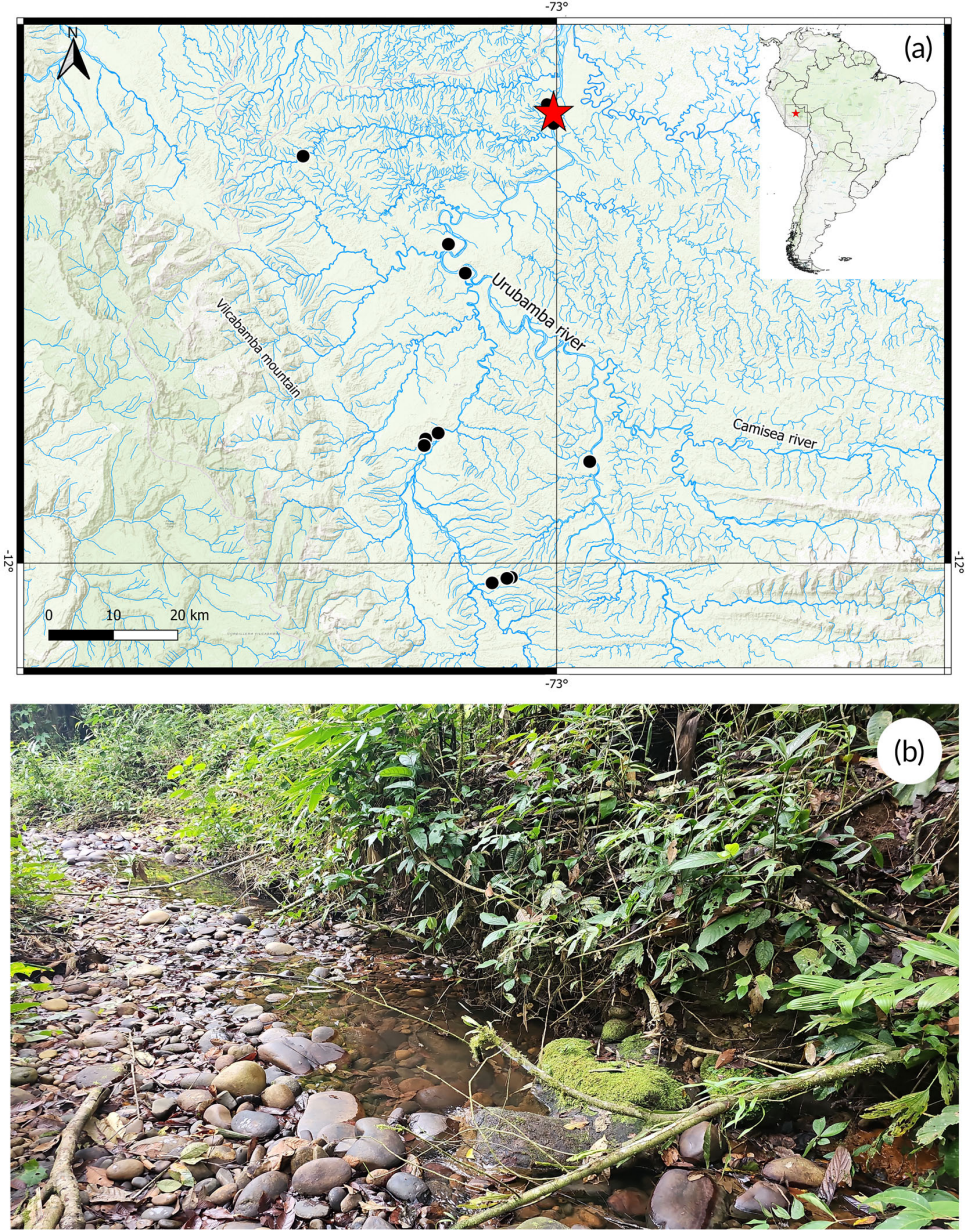
(a) Distribution of *Characidium ortegai*, with the type locality indicated by a red star and additional sampled localities represented by black circles (each symbol may correspond to more than one locality); (b) sampling locality of *C. ortegai* (MUSM 77986), Charapa Creek, a tributary of the Urubamba River, in the Megantoni District, La Convención Province, Amazon River basin.

*C. ortegai* is known from the main course of the Urubamba River and some of its major tributaries, including the Picha, Parotori and Miaria rivers, within the Ucayali River sub‐basin, Amazon basin, in southeastern Peru (Figure 3).

### 3.9 Ecological notes

*C. ortegai* inhabits small‐ to medium‐sized streams and creeks at elevations ranging from the Andean foothills to montane regions. Andean tropical montane forests (TMF) are hotspots of biodiversity that provide fundamental hydrological services as well as carbon sequestration and storage (Aragón et al., 2021). These watercourses are characterized by clear waters with seasonal flow variation, maintaining deeper pools during the rainy season (September–April) and becoming shallow with exposed rocky substrate during the dry season (May–August) (Fernández et al., 2020). The substrate typically consists of a heterogeneous mixture of pebbles, gravel and boulders, providing numerous microhabitats and refugia. The riparian vegetation reflects the transitional nature between Amazonian lowlands and Andean slopes, featuring semi‐dense canopy cover with characteristic montane and cloud forest elements that provide partial shading and contribute with organic matter to the aquatic ecosystem (Figure 3b). The species co‐occurs with *Characidium* aff. *purpuratum*, *Nemuroglanis* sp., *Rhamdia* sp., *Chrysobrycon myersi* (Weitzman & Thomerson), *Moenkhausia grandisquamis* (Müller & Troschel), *Ancistrus jelskii* (Steindachner) and *Ancistrus bufonius* (Valenciennes).

### 3.10 Conservation status

The available evidence possibly indicates that *C. ortegai* maintains a stable population across its known range, with repeated records over time and no signs of significant decline or fragmentation. The species occurs in structurally complex, well‐preserved habitats that support diverse fish assemblages and show no indication of severe or widespread anthropogenic disturbance. Given the absence of identified threats likely to cause rapid population reduction or range contraction, *C. ortegai* does not meet the criteria for any of the threatened categories. In accordance with the IUCN Red List Categories and Criteria, as outlined by the IUCN Standards and Petitions Committee (2024), the species is therefore proposed to be assessed as Least Concern (LC).

### 3.11 Etymology

The specific name *ortegai* is given in honour of ichthyologist Hernán Ortega Torres, for his significant contributions to the knowledge of the ichthyofauna of Peru and for being among the first to collect specimens of the species described herein.

### 3.12 Molecular analysis

**FIGURE 4:**
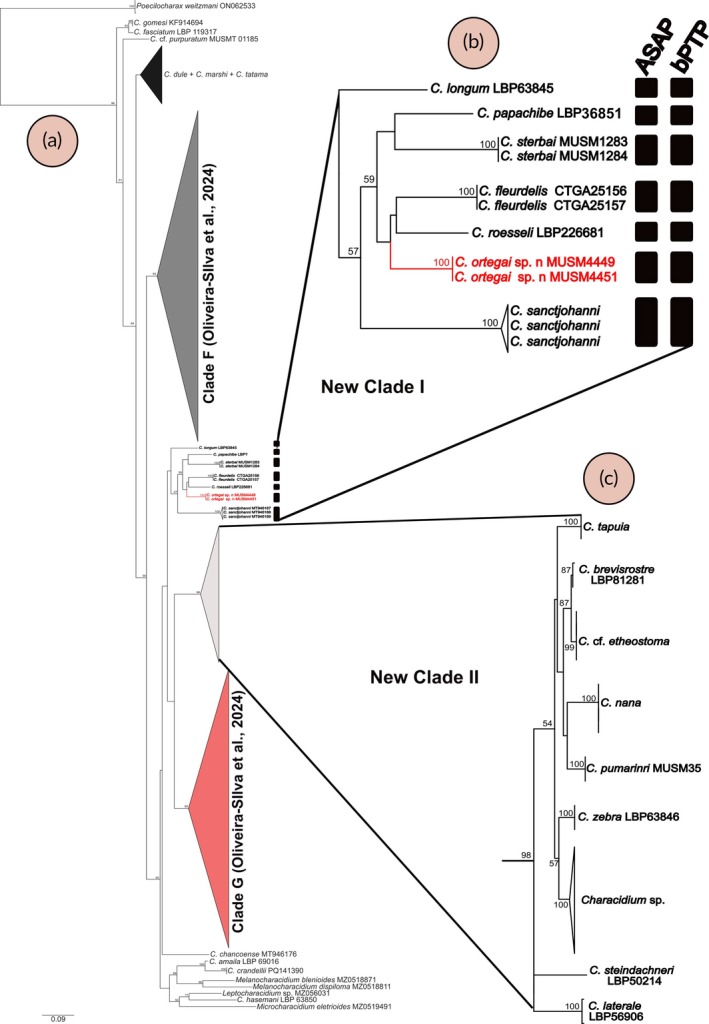
Maximum likelihood tree of species of *Characidium* inferred from cytochrome c oxidase subunit I (coI) gene sequences. Black vertical bars represent species delimitated by the Assemble Species by Automatic Partitioning (ASAP) and Poisson Tree Process (PTP) analyses. Bootstrap values above 50% are represented by numbers near the nodes. (a) Overall phylogenetic hypothesis proposed; (b) detailed view highlighting the relationships recovered within New Clade I; (c) detailed view highlighting the relationships recovered within New Clade II.

The molecular dataset comprised 156 sequences with 637 base pairs and 267 variable sites. The Iss values were lower than the corresponding critical values (Iss.c), indicating no evidence of substitution saturation in the dataset. Most of the clades recovered in the phylogenetic analysis (Figure 4a) are consistent with previous hypotheses proposed in the literature (see Discussion). However, our results revealed two distinct clades that were predominantly group species that had not been previously included in molecular phylogenetic analyses. These are herein referred to as New Clade I and New Clade II, to facilitate the interpretation and discussion of our results (Figure 4b,c). New Clade I, of main interest in this study, comprises *C. ortegai* along with *C. fleurdelis*, *C. longum*, *C. papachibe*, *C. sanctjohanni* Dahl and *C. sterbai*. Within the clade, *C. ortegai* is recovered as the sister group to *C. fleurdelis* + *C. roesseli*. Among the species of the clade, only *C. sanctjohanni* had been included previously in molecular phylogenetic studies. The ML tree recovered high bootstrap values supporting each of the analysed species in New Clade I (Figure 4b). The best partition proposed by ASAP identified seven MOTUs within the clade (ASAP score: 3.00), supporting *C. ortegai* as a distinct species. Remarkably, the bPTP analysis based on the ML tree recovered the same species hypotheses within the clade (Figure 4b). The interspecific genetic distance values within New Clade I ranged from 10% between *C. ortegai* and *C. fleurdelis*, to 16% between *C. sanctjohanni* and *C. longum* (Table 2). Regarding New Clade II, it is composed of some species previously included in molecular phylogenetic studies, such as *Characidium nana* Mendonça & Netto‐Ferreira, *C*. cf. *etheostoma*, *C. steindachneri*, *C. tapuia* Zanata, Ramos & Oliveira‐Silva and *C. zebra*, in addition to the newly included *C. brevirostre*, *C. pumarinri* and *C. laterale*.

**TABLE 2:** Genetic distances for the cytochrome oxidase c subunit 1 (coI) gene and standard errors of the *Characidium* species analysed in this study, belonging to the New Clade I, based on the p‐distance model.

	1	2	3	4	5	6
1. *Characidium fleurdelis*						
2. *Characidium longum*	0.13 ± 0.01					
**3.** ***Characidium ortegai***	**0.11 ± 0.01**	**0.14 ± 0.01**				
4. *Characidium papachibe*	0.12 ± 0.01	0.14 ± 0.01	0.12 ± 0.01			
5. *Characidium roesseli*	0.11 ± 0.01	0.12 ± 0.01	0.10 ± 0.01	0.12 ± 0.01		
6. *Characidium sanctjohanni*	0.13 ± 0.01	0.16 ± 0.01	0.13 ± 0.01	0.14 ± 0.01	0.14 ± 0.01	
7. *Characidium sterbai*	0.12 ± 0.01	0.13 ± 0.01	0.12 ± 0.01	0.12 ± 0.01	0.12 ± 0.01	0.13 ± 0.01

*Note*: The new species in bold.

## 4 DISCUSSION

*Characidium ortegai* is strongly supported by both morphological and molecular evidence, based on distinctive features such as an incomplete lateral line and unique sexual dichromatism, allied to interspecific genetic distances ranging from 10% to 16% compared to related species. Our results also corroborate earlier studies that recovered *Characidium* as not monophyletic, a pattern repeatedly reported in phylogenetic analyses based on molecular data (e.g., Oliveira et al., 2011; Oliveira‐Silva et al., 2024). These congruent findings reinforce the need for a comprehensive taxonomic revision of the genus, as current species assignments do not reflect evolutionary relationships accurately. However, our analysis also revealed novel molecular phylogenetic patterns that expand the current understanding of *Characidium* evolution and biogeography. Notably, two clades, herein referred to as New Clade I and New Clade II, predominantly group species were included for the first time in molecular studies. Given the preliminary nature of these findings and the characteristics of the marker used, which is not suitable for deep phylogenetic inferences (Morón‐López et al., 2022), further testing of the hypothesis with additional genomic approaches is recommended.

In the newly proposed Clade I, only *C. sanctjohanni* had been included in previous molecular studies (e.g., Agudelo‐Zamora et al., 2020). In Oliveira‐Silva et al. (2024), sequences of this species were recovered as sister to Clade B, which includes most species of *Characidium* in that study, without designating a specific name for the clade formed solely by *C. sanctjohanni*. Our results show that *C. sanctjohanni*, a trans‐Andean species from the San Juan River basin (Fricke et al., 2025), belongs to a newly proposed clade. Interestingly, this clade also includes cis‐Andean species from the Amazon basin, such as the new species described herein. Furthermore, most species within this clade (i.e., *C. fleurdelis*, *C. longum*, *C. papachibe* and *C. sterbai*) have previously been reported in the literature as possessing, or sharing, morphological traits with psammophilous species (e.g., Peixoto & Wosiacki, 2013; Zanata et al., 2023).

Psammophilous species are typically associated with environments characterized by loose, shifting sand and minimal structural complexity, requiring specific morphological and behavioural adaptations for substrate interaction and concealment (Buckup, 1993b; Henschel et al., 2020). Within *Characidium*, these species have been historically recognized by a suite of features including slender bodies and a pigmentation pattern formed by narrow, vertically oriented bars that are often fragmented, giving a spotted appearance (Buckup, 1993b). In his hypothesis, Buckup (1993b) associated these morphological patterns with a monophyletic assemblage corresponding to Clades C5–C7, which exhibit a nested series of synapomorphies involving progressive modifications of the vertical pigmentation, from a reduction in bar thickness (clade C5, composed by *C. pellucidum, C. pteroides, C. steindachneri and C*. sp. ‘*Urumari*’) to extremely thin lines seldom wider than one scale (clade C6, composed by *C. pellucidum, C. pteroides and C*. sp. *‘Urumari’*), and ultimately to fragmented, posteriorly curved marks disconnected from their dorsal origins (clade C7, composed by *C. pellucidum and C. pteroides*). These pigmentation traits were interpreted by the author as part of a transformation series likely linked to ecological adaptation to sandy substrates, as most species assigned to these clades are found in such habitats. However, our findings suggest that these traits may not be exclusive to a single evolutionary lineage. For instance, *C. steindachneri*, traditionally considered psammophilous, was recovered within New Clade II, phylogenetically distant from other psammophilous species recovered within New Clade I, and casting doubt on the monophyly of the psammophilous assemblage as previously hypothesized.

The newly proposed Clade I is composed, as stated previously, of species that possess or share morphological traits with psammophilous species. However, *C. ortegai* does not conform to the ecological or morphological characteristics typically associated with psammophily. It inhabits small‐ to medium‐sized streams and creeks in tropical montane forest environments, where substrates are heterogeneous and composed of pebbles, gravel and boulders rather than sand. These habitats, situated within semi‐shaded riparian zones of transitional Andean‐Amazonian forest, offer structurally complex microhabitats, unlike the simplified sandy environments associated with psammophily. Morphologically, *C. ortegai* also lacks key traits shared by psammophilous species, such as a complete lateral line and an adipose fin, features consistently present in *C. longum*, *C. papachibe*, *C. pellucidum*, *C. pteroides* and *C. steindachneri*. Future studies incorporating broader taxon sampling, ecological data and genomic approaches will be essential to clarify the evolutionary origins and significance of psammophily in *Characidium*.

With the addition of *C. ortegai*, the number of species of *Characidium* recorded from the Ucayali‐Urubamba Piedmont ecoregion rises to nine. This new record complements the list previously documented by Chuctaya et al. (2022), which includes *C. etheostoma*, *C. geryi*, *C. pteroides*, *C. purpuratum*, *C. roesseli*, *C. steindachneri*, *C. sterbai* and *C. zebra*. The occurrence of *C. ortegai* reinforces the remarkable species richness of *Characidium* in Andean piedmont drainages, a region characterized by high habitat heterogeneity and complex hydrological networks (Benejam et al., 2018), which may promote both ecological specialization and allopatric diversification. This finding not only expands the known distribution of the genus but also highlights the relevance of reexamining specimens deposited in scientific collections, where previously unidentified or newly recognized diversity may be documented. Continued taxonomic revisions and targeted field surveys in underexplored areas remain crucial for revealing the full extent of Neotropical fish species richness and the evolutionary mechanisms underlying it.

## 5 COMPARATIVE MATERIAL EXAMINED

Comparative material was obtained from the list of species provided by Zanata et al. (2018, 2023), with the addition of *C. geryi* MUSM 21873, 10, 13.6–19.5 mm *L*_*S*_, 1 c&s, 17.9 mm *L*_*S*_, Marañón River basin; MUSM 41993, 1, 25.8 mm *L*_*S*_, Marañón River basin. *C*. cf. *pellucidum* MZUSP 118834, 25, 17.4–23.0 mm *L*_*S*_, Madeira River basin; MZUSP 121994, 7, 26.1–28.9 mm *L*_*S*_, Madeira River basin. *C. schindleri* Zarske & Géry AMNH 233322, 10, 40.1–65.3 mm *L*_*S*_, Madeira River basin. *C. sterbai* UFBA 10825, 6, 24.6–30.7 mm *L*_*S*_, Ucayali River basin.

## AUTHOR CONTRIBUTIONS

Conceptualization: Leonardo Oliveira‐Silva and Angela Maria Zanata. Developing methods: Leonardo Oliveira‐Silva and Angela Maria Zanata. Conducting the research, data analysis and data interpretation: all authors. Writing: all authors. Funding: all authors.

## FUNDING INFORMATION

This work was financed by FAPESP (grant 2023/09871‐6) (LOS), which also provided a postdoctoral fellowship supporting the analysis of the fish collection at the Natural History Museum of the National University of San Marcos (Lima, Peru). Claudio Oliveira received financial support from the São Paulo Research Foundation (FAPESP grant 2020/13433‐6), the Brazilian National Council for Scientific and Technological Development (CNPq grants 306054/2006‐0 and 441128/2020‐3) and the Office of the Vice President for Research at São Paulo State University (Prope‐UNESP). Also the work was financed by CONCYTEC through the PROCIENCIA programme within the framework of the contest [PE501084299‐2023‐PROCIENCIA‐BM] within the framework of the call ‘Interinstitutional Alliances for Doctorate Programs’ (VMV), [PE501085130‐2023‐PROCIENCIA] (DRFF) and [PE501082404‐2023‐PROCIENCIA] within the framework of the call ‘2023‐01 Basic Research Project’ (RB). The Article Processing Charge for the publication of this research was funded by the Coordenação de Aperfeiçoamento de Pessoal de Nível Superior‐Brasil (CAPES) (ROR identifier: 00x0ma614).

## CONFLICT OF INTEREST STATEMENT

This study was done without conflict of interest.

## Supporting information


**Table S1.** Taxa, vouchers, locality and GenBank accession numbers of specimens of *Characidium* used in the mitochondrial DNA analysis. The acronyms of institutions follow Fricke and Eschmeyer (2025).
