# Supplementary material for: Morphological and molecular evidence reveals a new species of Characidium from the Ucayali‐Urubamba Piedmont, Peru, and novel molecular clades are proposed within the genus
Source: J Fish Biol. 2025 Nov 6;108(2):584–95. doi: 10.1111/jfb.70261 (PMC13052454; doi:10.1111/jfb.70261)
Supplement: Supplementary file 1 — Table S1. Taxa, vouchers, locality and GenBank accession numbers of specimens of Characidium used in the mitochondrial DNA analysis. The acronyms of institutions follow Fricke and Eschmeyer (2025). [file JFB-108-584-s001.docx]

**Table S1**. Taxa, vouchers, locality and GenBank accession numbers of specimens of *Characidium* used in the mitochondrial DNA analysis. The acronyms of institutions follow Fricke & Eschmeyer (2025).

| Taxon | Museum ID | Specimen | Locality | City, State | Country | GenBank n. |
| --- | --- | --- | --- | --- | --- | --- |
| *Crenuchus spilurus* | LBP 4275 | LBP 23869 | Rio Negro - Amazônica | Barcelos, Amazonas, BR | Brazil | KF914693 |
| *Leptocharacidium sp.* | - | SU08-1265-2 | Maroni river | Paloemeu river, Sipaliwini | Suriname | MZ051603 |
| *Microcharacidium eleotrioides* | - | GFSU14e-1530-2 | Litany river | Upper Maroni | French Guiana | MZ051949 |
| *Melanocharacidium blennioides* | - | SU08-228 | Maroni river | Paloemeu river, Sipaliwini | Suriname | MZ051887 |
| *Melanocharacidium dispilomma* | - | SU08-1255-1 | Maroni river | Tapanahony River, Sipaliwini | Suriname | MZ051881 |
| *Poecilocharax weitzmani* | INPA-ICT 042889 | P29425 | Rio Negro - Amazônica | São Gabriel da Cachoeira, Amazonas | Brazil | ON062533.1 |
| *Poecilocharax weitzmani* | INPA-ICT 042889 | P29426 | Rio Negro - Amazônica | São Gabriel da Cachoeira, Amazonas | Brazil | ON063012.1 |
| *Poecilocharax weitzmani* | INPA-ICT 042889 | P29427 | Rio Negro - Amazônica | São Gabriel da Cachoeira, Amazonas | Brazil | ON063016.1 |
| *Characidium alipioi* A | LBP 25740 | 78709 | Rio Paraíba do Sul | Pindamonhangaba, São Paulo | Brazil | MH716095 |
| *Characidium alipioi* A | LBP 25740 | 78712 | Rio Paraíba do Sul | Pindamonhangaba, São Paulo | Brazil | MH716097 |
| *Characidium alipioi* A | LBP 25740 | 78713 | Rio Paraíba do Sul | Pindamonhangaba, São Paulo | Brazil | MH716098 |
| *Characidium alipioi* A | LBP 25740 | 78750 | Rio Paraíba do Sul | Pindamonhangaba, São Paulo | Brazil | MH716119 |
| *Characidium alipioi* B | LBP 25739 | 78711 | Rio Paraíba do Sul | Pindamonhangaba, São Paulo | Brazil | MH716122 |
| *Characidium alipioi* B | LBP 25739 | 78714 | Rio Paraíba do Sul | Pindamonhangaba, São Paulo | Brazil | MH716123 |
| *Characidium alipioi* B | LBP 25739 | 78717 | Rio Paraíba do Sul | Pindamonhangaba, São Paulo | Brazil | MH716124 |
| *Characidium alipioi* B | LBP 25739 | 78726 | Rio Paraíba do Sul | Pindamonhangaba, São Paulo | Brazil | MH716127 |
| *Characidium alipioi* B | LBP 25739 | 78728 | Rio Paraíba do Sul | Pindamonhangaba, São Paulo | Brazil | MH716128 |
| *Characidium amaila* | LBP 17445 | 69016 | Potaro river | Potaro-Siparuni | French Guiana | PX210808 |
| *Characidium bahiense* | UFBA 9072 | 2378 | Rio Capivara Grande | Camaçari, Bahia | Brazil | OQ998773 |
| *Characidium bahiense* | UFBA 9072 | 2379 | Rio Capivara Grande | Camaçari, Bahia | Brazil | OQ998774 |
| *Characidium bahiense* | UFBA 9072 | 2380 | Rio Capivara Grande | Camaçari, Bahia | Brazil | OQ998775 |
| *Characidium bahiense* | UFBA 9072 | 2381 | Rio Capivara Grande | Camaçari, Bahia | Brazil | OQ998776 |
| *Characidium bahiense* | UFBA 9072 | 2382 | Rio Capivara Grande | Camaçari, Bahia | Brazil | OQ998777 |
| *Characidium bimaculatum* | UFRN 5628 | 5384 | Rio Paraíba do Norte | São Miguel de Itaipu, Paraíba | Brazil | PP725048 |
| *Characidium bimaculatum* | UFRN 5628 | 5385 | Rio Paraíba do Norte | São Miguel de Itaipu, Paraíba | Brazil | PP725049 |
| *Characidium bimaculatum* | UFRN 5628 | 5386 | Rio Paraíba do Norte | São Miguel de Itaipu, Paraíba | Brazil | PP725050 |
| *Characidium bimaculatum* | UFRN 5628 | 5387 | Rio Paraíba do Norte | São Miguel de Itaipu, Paraíba | Brazil | PP725051 |
| *Characidium bimaculatum* | UFRN 5658 | 5388 | Mamanguape | Alagoa Nova, Paraíba | Brazil | PP725052 |
| *Characidium brevirostre* | LBP 21033 | 81280 | Rio Calçoene | Calçone, Amapá | Brazil | PX210809 |
| *Characidium brevirostre* | LBP 21033 | 81281 | Rio Calçoene | Calçone, Amapá | Brazil | PX210810 |
| *Characidium cacah* | MCNIP4630 | 4452 | Rio São Francisco | Jaboticatubas, Minas Gerais | Brazil | OQ998801 |
| *Characidium cacah* | MCNIP4633 | 4457 | Rio São Francisco | Jaboticatubas, Minas Gerais | Brazil | PP725095 |
| *Characidium cacah* | MCNIP4633 | 4458 | Rio São Francisco | Jaboticatubas, Minas Gerais | Brazil | PP725096 |
| *Characidium cacah* | UFBA 4459 | 4453 | Rio São Francisco | Jaboticatubas, Minas Gerais | Brazil | PP725097 |
| *Characidium chancoense* |  | CHIFF0016 | Magdalena-Cauca | Mediacanoa alto Cauca | Colômbia | MT946176 |
| *Characidium clistense* | UFBA 7059 | 2461 | Rio Paraguaçu | Lençóis, Bahia | Brazil | OQ998803 |
| *Characidium clistense* | UFBA 7059 | 2462 | Rio Paraguaçu | Lençóis, Bahia | Brazil | PP725098 |
| *Characidium clistense* | UFBA 7059 | 2463 | Rio Paraguaçu | Lençóis, Bahia | Brazil | PP725099 |
| *Characidium crandellii* | LBP 7070 | 34389 | Rio Negro | São Gabriel da Cachoeira, Amazonas | Brazil | PQ141390 |
| *Characidium crandellii* | LBP 7070 | 34390 | Rio Negro | São Gabriel da Cachoeira, Amazonas | Brazil | PQ141391 |
| *Characidium cricarense* | MNRJ 50973 | 8269 | Rio São Mateus | São Mateus, Espírito Santo | Brazil | MH667896 |
| *Characidium cricarense* | MNRJ 41832 | 5314 | Rio Doce | Santa Teresa, Espírito Santo | Brazil | MH667899 |
| *Characidium cricarense* | MNRJ 41832 | 5315 | Rio Doce | Santa Teresa, Espírito Santo | Brazil | MH667878 |
| *Characidium cricarense* | MNRJ 41832 | 5315 | Rio Doce | Santa Teresa, Espírito Santo | Brazil | MH667864 |
| *Characidium cricarense* | CZNC 1506 | 8162 | Rio São Mateus | Água Doce do Norte, Espírito Santo | Brazil | MH667902 |
| *Characidium deludens* | UFBA 8432 | 2454 | Rio Paraguaçu | Piatã, Bahia | Brazil | PP725101 |
| *Characidium deludens* | UFBA 8432 | 2455 | Rio Paraguaçu | Piatã, Bahia | Brazil | PP725102 |
| *Characidium deludens* | UFBA 8432 | 2456 | Rio Paraguaçu | Piatã, Bahia | Brazil | PP725103 |
| *Characidium dule* | IMCN 8929 | 8929 | Rio Atrato | - | Colômbia | MT946177 |
| *Characidium dule* | IMCN 8929 | 8929 | Rio Atrato | - | Colômbia | MT946178 |
| *Characidium dule* | IMCN 8929 | 8929 | Rio Atrato | - | Colômbia | MT946179 |
| *Characidium dule* | IMCN 8929 | 8929 | Rio Atrato | - | Colômbia | MT946180 |
| *Characidium* cf. *etheostoma* | LBP 9286 | 43760 | Rio Guamá | Ourém, Pará | Brazil | PQ141392 |
| *Characidium* cf. *etheostoma* | LBP 9286 | 43761 | Rio Guamá | Ourém, Pará | Brazil | PQ141393 |
| *Characidium fasciatum* | LBP 36852 | 119317 | Rio São Francisco | Itapecerica, Minas Gerais | Brazil | PX210811 |
| *Characidium fleurdelis* | - | CTGA 25156 | Rio Guaporé | Corumbiara, Rondônia | Brazil | PX210812 |
| *Characidium fleurdelis* | - | CTGA 25157 | Rio Guaporé | Corumbiara, Rondônia | Brazil | PX210813 |
| *Characidium* cf. *etheostoma* | LBP 9286 | 43762 | Rio Guamá | Ourém, Pará | Brazil | PQ141394 |
| *Characidium hasemani* | LBP 15509 | 63850 | Rio Takutu-Amazonas | Bomfim, Roraima | Brazil | PX210814 |
| *Characidium helmeri* | MZUSP 112653 | 587 | Rio Cahy | Prado, Bahia | Brazil | PP725112 |
| *Characidium helmeri* | MZUSP 112666 | 585 | Rio Cahy | Prado, Bahia | Brazil | PP725111 |
| *Characidium helmeri* | UFBA 8715 | 2504 | Rio Cahy | Prado, Bahia | Brazil | PP725113 |
| *Characidium* cf. *interruptum* | UFBA 8711 | 2490 | Rio Cahy | Prado, Bahia | Brazil | PP725110 |
| *Characidium* cf. *interruptum* | UFBA 8709 | 2485 | Rio Cahy | Prado, Bahia | Brazil | PP725108 |
| *Characidium* cf. *interruptum* | UFBA 8709 | 2486 | Rio Cahy | Prado, Bahia | Brazil | PP725109 |
| *Characidium interruptum* | UFBA 10006 | 2874 | Rio São João | Silva Jardim, Rio de Janeiro | Brazil | PQ141395 |
| *Characidium interruptum* | UFBA 10006 | 2875 | Rio São João | Silva Jardim, Rio de Janeiro | Brazil | PQ141396 |
| *Characidium itarare* | - | NUP21084 | Upper Rio Parana | Jaguariaiva, Parana | Brazil | OQ749887 |
| *Characidium itarare* | - | NUP21063 | Upper Rio Parana | Jaguariaiva, Parana | Brazil | OQ749888 |
| *Characidium kamakan* | UFBA 8765 | 2708 | Rio Pardo | Camacã, Bahia | Brazil | PP725114 |
| *Characidium kamakan* | UFBA 8765 | 2709 | Rio Pardo | Camacã, Bahia | Brazil | PP725115 |
| *Characidium kamakan* | UFBA 8765 | 2710 | Rio Pardo | Camacã, Bahia | Brazil | PP725116 |
| *Characidium krenak* | - | LGC 5752 | Rio Doce | Conceição do Mato Dentro, Minas Gerias | Brazil | BOLD:ACS9348 |
| *Characidium krenak* | - | LGC 5719 | Rio Doce | Alvorada de Minas, Minas Gerais | Brazil | BOLD:ACS9348 |
| *Characidium krenak* | MCNIP 4889 | LGC RD161 | Rio Doce | Conceição do Mato Dentro, Minas Gerias | Brazil | BOLD:ACS9348 |
| *Characidium krenak* | LGC 5742 | LGC 5742 | Rio Doce | Bom Despacho, Minas Gerais | Brazil | BOLD:ACS9348 |
| *Characidium lanei* | LBP 7888 | 37048 | Rio Indaiá | Ubatuba, São Paulo | Brazil | PQ141397 |
| *Characidium lanei* | LBP 7888 | 37049 | Rio Indaiá | Ubatuba, São Paulo | Brazil | PQ141398 |
| *Characidium lanei* | LBP 7888 | 37050 | Rio Indaiá | Ubatuba, São Paulo | Brazil | PQ141399 |
| *Characidium lanei* | LBP 7888 | 33730 | Rio Indaiá | Ubatuba, São Paulo | Brazil | KF914702 |
| *Characidium lanei* | LBP 8700 | 33505 | Rio Morumbi | Morretes, Paraná | Brazil | KF914697 |
| *Characidium lanei* | MNRJ 40905 | 4970 | Guaraguaçu | Paranaguá, Paraná | Brazil | MG825016 |
| *Characidium* cf. *lanei* | LBP 5039 | 25983 | Rio Piraí | Joinville, Santa Catarina | Brazil | PX210815 |
| *Characidium* cf. *lanei* | LBP 5039 | 25984 | Rio Piraí | Joinville, Santa Catarina | Brazil | PX210816 |
| *Characidium laterale* | LBP 13709 | 56906 | Rio Paraguai | Paconé, Mato Grosso | Brazil | PX210817 |
| *Characidium laterale* | LBP 13709 | 56908 | Rio Paraguai | Paconé, Mato Grosso | Brazil | PX210818 |
| *Characidium litorale* | MNRJ 47087 | 11592 | Rio São Joao | Saquarema, Rio de Janeiro | Brazil | MH667873 |
| *Characidium longum* | LBP 5507 | 163845 | Rio Takutu, Amazonas | Bonfim, Roraima | Brazil | PX210819 |
| *Characidium marshi* | STRI 01035 | 3611 | Rio Ipeti | Ipeti | Panamá | MG936837 |
| *Characidium marshi* | STRI 01037 | 4069 | Rio Tuira | Tuira | Panamá | MG936836 |
| *Characidium marshi* | STRI 01042 | 6730 | Rio Tuira | Tuira | Panamá | MG936834 |
| *Characidium nana* | LBP 25215 | 94142 | Rio Xingu | Altamira, Pará | Brazil | PQ141400 |
| *Characidium nana* | LBP 25215 | 94143 | Rio Xingu | Altamira, Pará | Brazil | PQ141401 |
| *Characidium nana* | LBP 25215 | 94144 | Rio Xingu | Altamira, Pará | Brazil | PQ141402 |
| *Characidium nupelia* | LBP 35592 | 116662 | Upper Paraguai | Rosário do Oeste, Mato Grosso | Brazil | PX210820 |
| *Characidium nupelia* | LBP 35592 | 116663 | Upper Paraguai | Rosário do Oeste, Mato Grosso | Brazil | PX210821 |
| *Characidium occidentale* | LBP 19562 | 62712 | Rio Uruguai | São Nicolau, Rio Grande do Sul | Brazil | PX210822 |
| *Characidium oiticicai* | LBP 2927 | 18793 | Rio Tietê | Salesópolis, São Paulo | Brazil | PX210823 |
| *Characidium orientale* | MCP 48744 | 21188 | Rio das Antas | Jaquirana, Rio Grande do Sul | Brazil | PX210824 |
| *Characidium ortegai* | MUSM 77986 | MUSMT04449 | Rio Urubamba | La Convenciçón, Cusco | Peru | PX210825 |
| *Characidium ortegai* | MUSM 77986 | MUSMT04451 | Rio Urubamba | La Convenciçón, Cusco | Peru | PX210826 |
| *Characidium papachibe* | LBP 36851 | 36851 | Arapiuns, Amazonas | Santarém, Pará | Brazil | PX210827 |
| *Characidium pterostictum* | LBP 8700 | 33509 | Atlantic coastal river | Morretes, Paraná | Brazil | KF914710 |
| *Characidium pterostictum* | LBP 8701 | 33516 | Atlantic coastal river | Paranaguá, Paraná | Brazil | KF914709 |
| *Characidium pterostictum* | LBP 7367 | 33636 | Rio Ribeira de Iguape | Iporanga, São Paulo | Brazil | KF914700 |
| *Characidium pterostictum* | LBP 7367 | 33671 | Rio Ribeira de Iguape | Iporanga, São Paulo | Brazil | KF914708 |
| *Characidium pumarinri* | MUSM 74149 | LGBBF03528 | Río Monzón | Tingo María, Huánuco | Peru | PX210828 |
| *Characidium pumarinri* | MUSM 74149 | LGBBF03529 | Río Monzón | Tingo María, Huánuco | Peru | PX210829 |
| *Characidium* cf. *purpuratum* | MUSM 78354 | MUSMT01185 | Río Aguaytía | Padre Abad, Ucayali | Peru | PX210830 |
| *Characidium rachovii* | LBP 3359 | 21188 | Arrio Bolacha | Rio Grande, Rio Grande do Sul | Brazil | PX210831 |
| *Characidium rachovii* | LBP 3359 | 21189 | Arrio Bolacha | Rio Grande, Rio Grande do Sul | Brazil | PX210832 |
| *Characidium sanctjohanni* | IMCN 8928 | - | Rio Opogodó | - | Colômbia | MT946187 |
| *Characidium sterbai* | MUSM 74147 | MUSMT01283 | Río Shambo | Boquerón, Ucayali | Peru | PX210833 |
| *Characidium sterbai* | MUSM 74147 | MUSMT01284 | Río Shambo | Boquerón, Ucayali | Peru | PX210834 |
| *Characidium roesseli* | LBP 22668 | 22668 | Rio Amazonas | Tabatinga, Amazonas | Brazil | PX210835 |
| *Characidium sanctjohanni* | IMCN 8928 | - | Rio Opogodó | - | Colômbia | MT946188 |
| *Characidium sanctjohanni* | IMCN 8928 | - | Rio Opogodó | - | Colômbia | MT946189 |
| *Characidium samurai* | UFBA 8712 | 2491 | Rio das Almas | Piraí do Norte, Bahia | Brazil | PP725117 |
| *Characidium samurai* | MZUSP 112385 | 594 | Rio das Almas | Piraí do Norte, Bahia | Brazil | PP725118 |
| *Characidium satoi* | LBP 24066 | 92056 | Rio São Francisco | São Gonçalo do Abaeté, Minas Gerais | Brazil | PP725119 |
| *Characidium satoi* | LBP 24066 | 92057 | Rio São Francisco | São Gonçalo do Abaeté, Minas Gerais | Brazil | OQ998802 |
| *Characidium satoi* | LBP 24066 | 92058 | Rio São Francisco | São Gonçalo do Abaeté, Minas Gerais | Brazil | PP725120 |
| *Characidium schubarti* | LBP 8702 | 31498 | Upper Rio Paraná | Jaguariaíva, Paraná | Brazil | GU701439 |
| *Characidium schubarti* | LBP 8702 | 31513 | Upper Rio Paraná | Jaguariaíva, Paraná | Brazil | GU701442 |
| *Characidium schubarti* | LBP 8702 | 31526 | Upper Rio Paraná | Jaguariaíva, Paraná | Brazil | GU701441 |
| *Characidium steindachneri* | LBP 10895 | 50214 | Rio Madeira | Porto Velho, Rondônia | Brazil | PX210836 |
| *Characidium tapuia* | LBP 5561 | 27263 | Rio Parnaíba | Santa Filomena, Piauí | Brazil | PQ141411 |
| *Characidium tapuia* | LBP 5561 | 27264 | Rio Parnaíba | Santa Filomena, Piauí | Brazil | PQ141412 |
| *Characidium tatama* | IMCN 8926 | 8926 | Rio San Juan | - | Colômbia | MT946199 |
| *Characidium tatama* | IMCN 8927 | 8927 | Rio San Juan | - | Colômbia | MT946200 |
| *Characidium tenue* | LBP14514 | 60772 | Arroio Chuí | Chuy, Rio Grande do Sul | Brazil | PX210837 |
| *Characidium tenue* | LBP14514 | 60773 | Arroio Chuí | Chuy, Rio Grande do Sul | Brazil | PX210838 |
|  |  |  |  |  |  |  |
| *Characidium timbuiense* | UFBA 9938 | 3602 | Reis Magos | Santa Teresa, Espírito Santo | Brazil | PQ141387 |
| *Characidium timbuiense* | UFBA 9938 | 3603 | Reis Magos | Santa Teresa, Espírito Santo | Brazil | PQ141388 |
| *Characidium timbuiense* | UFBA 9938 | 3604 | Reis Magos | Santa Teresa, Espírito Santo | Brazil | PQ141389 |
| *Characidium timbuiense* | LBP 19565 | 69812 | Reis Magos | Santa Teresa, Espírito Santo | Brazil | KM229365 |
| *Characidium timbuiense* | MNRJ 41900 | 9077 | Reis Magos | Santa Teresa, Espírito Santo | Brazil | MH667892 |
| *Characidium travassosi* | MCP 22605 | 22605 | Rio Iguaçu | Candói, Paraná | Brazil | PX210839 |
| *Characidium varii* | LBP 32183 | 112651 | Teles Pires | Guarantã do Norte, Mato Grosso | Brazil | PQ141408 |
| *Characidium varii* | LBP 32183 | 112652 | Teles Pires | Guarantã do Norte, Mato Grosso | Brazil | PQ141409 |
| *Characidium varii* | LBP 32183 | 112654 | Teles Pires | Guarantã do Norte, Mato Grosso | Brazil | PQ141410 |
| *Characidium xanthopterum* | LBP 7254 | 34895 | Upper Rio Paraná | Pires do Rio, Goiás | Brazil | GU701836 |
| *Characidium xanthopterum* | LBP 7254 | 35896 | Upper Rio Paraná | Pires do Rio, Goiás | Brazil | GU701835 |
| *Characidium xanthopterum* | LBP 7254 | 34897 | Upper Rio Paraná | Pires do Rio, Goiás | Brazil | GU701834 |
| *Characidium xanthopterum* | LBP 7254 | 34898 | Upper Rio Paraná | Pires do Rio, Goiás | Brazil | GU701837 |
| *Characidium xanthopterum* | LBP 7286 | 35890 | Upper Rio Paraná | Caldas Novas, Minas Gerais | Brazil | GU701833 |
| *Characidium zebra* | LBP 15508 | 63846 | Rio Takuto, Amazonas | Bonfim, Roraima | Brazil |  |
| *Characidium zebra* | LBP 15508 | 63847 | Rio Takutu, Amazonas | Bonfim, Roraima | Brazil |  |
| *Characidium* sp. | LBP 25266 | 94361 | Rio Tapajós | Novo Progresso, Pará | Brazil | PQ141403 |
| *Characidium* sp. | LBP 25266 | 94362 | Rio Tapajós | Novo Progresso, Pará | Brazil | PQ141404 |
| *Characidium* sp. | LBP 25266 | 94363 | Rio Tapajós | Novo Progresso, Pará | Brazil | PQ141405 |
| *Characidium* sp. | LBP 25266 | 94364 | Rio Tapajós | Novo Progresso, Pará | Brazil | PQ141406 |
| *Characidium* sp. | LBP 25266 | 94365 | Rio Tapajós | Novo Progresso, Pará | Brazil | PQ141407 |
